# MBEC Versus MBIC: the Lack of Differentiation between Biofilm Reducing and Inhibitory Effects as a Current Problem in Biofilm Methodology

**DOI:** 10.1186/s12575-019-0106-0

**Published:** 2019-09-13

**Authors:** Lara Thieme, Anita Hartung, Kristina Tramm, Mareike Klinger-Strobel, Klaus D. Jandt, Oliwia Makarewicz, Mathias W. Pletz

**Affiliations:** 1 Institute for Infectious Diseases and Infection Control, Jena University Hospital, Am Klinikum 1, 07747 Jena, Germany; 2 Otto Schott Institute of Materials Research, Friedrich Schiller University Jena, Löbdergraben 32, Jena, 07743 Germany

**Keywords:** Biofilm-associated infections, Biofilm susceptibility testing, Biofilm susceptibility endpoint parameters, MBEC, MBIC

## Abstract

**Background:**

Biofilms are communities of aggregated, matrix-embedded microbial cells showing a high tolerance to an in principle adequate antibiotic therapy, often resulting in treatment failure. A major challenge in the management of biofilm-associated infections is the development of adequate, standardized biofilm susceptibility testing assays that are clinically meaningful, i.e. that their results correlate with treatment outcome. Different biofilm susceptibility endpoint parameters like the minimal biofilm eradication concentration (MBEC) or the minimal biofilm inhibitory concentration (MBIC) have been suggested as a guide for treatment of biofilm-associated infections, however with inconsistent perception and use among biofilm researchers, leading to confusion and contradictions among different anti-biofilm component studies and clinical trials.

**Findings:**

Evaluation of anti-biofilm effects is mostly based on the untreated reference growth control biofilm measured at the same endpoint as the treated biofilm, neglecting the possible change of the untreated reference biofilm from the time point of pre-antimicrobial exposure to the measured endpoint. In this commentary, we point out the importance of individual quantification of mature, established biofilms before antimicrobial treatment for each biofilm model in order to draw conclusions on the measured biofilm effect size, i.e. biofilm reducing (MBEC) or biofilm inhibitory (MBIC) effects.

**Conclusion:**

The assessment of pre-treatment biofilms contributes to a standardized use of biofilm susceptibility endpoint parameters, which is urgently needed to improve the clinical validity of future anti-biofilm assays.

## Background

Biofilms are matrix-embedded communities of microbial cells that are attached to each other and/or on a surface [1]. Biofilms protect enclosed bacterial cells against the immune system and an in principle adequate antibiotic therapy, often resulting in treatment failure, relapsing infections and increased mortality [1]. The minimal inhibitory concentrations (MIC) of antibiotics are routinely determined using planktonic bacteria and do not match the concentrations that are required to prevent, inhibit, diminish or eradicate biofilms [2].

A major challenge in the management of biofilm-associated infections (BAI) is the development of adequate, standardized biofilm susceptibility testing assays that are clinically meaningful, i.e. that their results correlate with treatment outcome [3, 4]. Over the last years, a multitude of diverse laboratory methods to assess anti-biofilm treatments has been developed. Each method has its own benefits and drawbacks as critically discussed elsewhere, with the overall consensus that there is currently no optimal biofilm method available mimicking the in vivo biofilm setting of human BAI [3, 5, 6]. Different biofilm susceptibility endpoint parameters have been suggested as a guide for treatment of BAI, like the minimal biofilm eradication concentration (MBEC), the minimal biofilm inhibitory concentration (MBIC), the biofilm bactericidal concentration (BBC) or the biofilm prevention concentration (BPC) [2]. However, the definition and interpretation of these parameters differ greatly among publications and none of the official agencies, e.g. EUCAST or CLSI, have yet set up standardized definitions of biofilm endpoint parameters likewise the MIC. While some researchers define the MBEC as the lowest concentration of an antimicrobial substance that eradicates 99.9% of biofilm-embedded bacteria (3 log_10_ reduction in CFU/mL) compared to growth controls [7], others define the former as the BBC in line with the minimal bactericidal concentration (MBC) on planktonic level and refer to the MBEC in the context of complete eradication of the biofilm [2, 8]. Inhibitory effects on biofilm formation are commonly assessed by the MBIC, which is the lowest concentration of an antimicrobial substance at which there is no time-dependent increase in the mean number of biofilm viable cells [2]. In contrast to the MBIC, the BPC determines at which antimicrobial substance concentration the cell density of a planktonic culture is sufficiently reduced in order to prevent biofilm formation [2].

In this commentary, we point out the importance of individual quantification of mature, established biofilms before antimicrobial treatment for each biofilm model in order to draw conclusions on the measured biofilm effect size, i.e. biofilm reducing or biofilm inhibitory effects.

## Findings

**Fig. 1:**
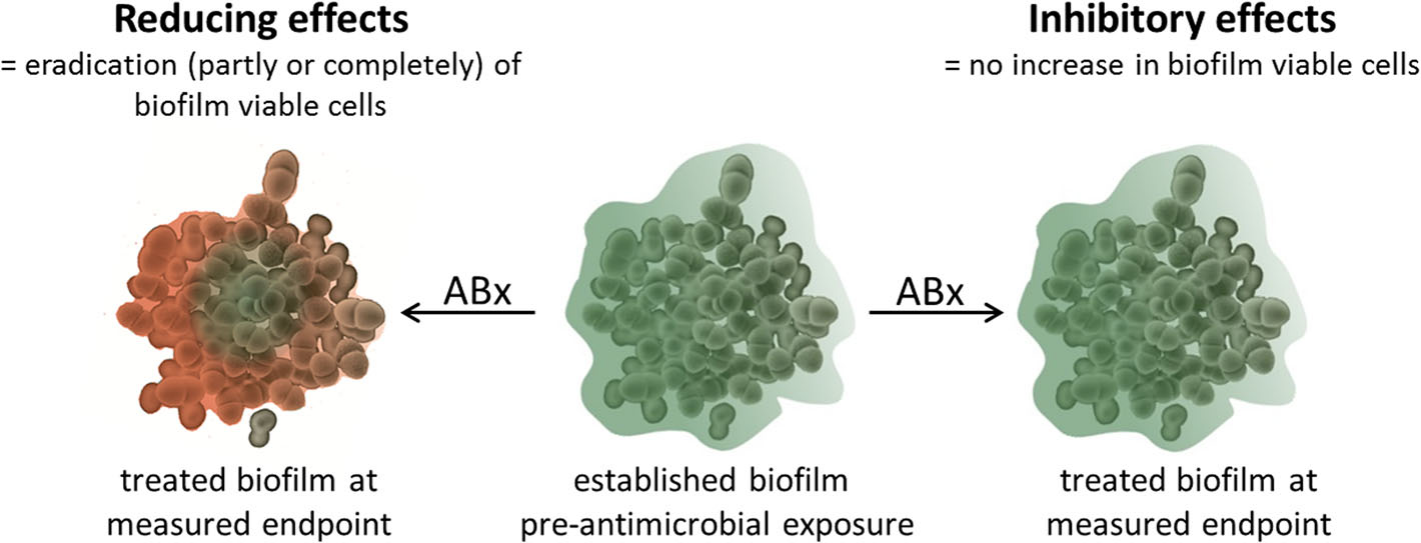
Reducing versus inhibitory effects on mature biofilms. Green indicates viable cells, red indicates dead cells. ABx = antimicrobial treatment

Importantly, all of the above parameters, except for the BPC, analyse the activity of antimicrobial substances on mature, established biofilms, so the experimental set-up to assess either biofilm reducing or inhibitory effects is in principle the same, regardless of the method of choice of biofilm growth and assay readout (Fig. 1). After establishment - while even the time of biofilm maturation varies strongly between different research groups -, biofilms are treated with the respective antimicrobial substance for a variable period of time (hours to days), followed by assessment of the treated and untreated biofilms by e.g. CFU/mL determination, image acquisition or staining and photometric measurement (e.g. resazurin or crystal violet). Evaluation of anti-biofilm effects is thereby mostly based on the untreated reference growth control biofilm measured at the same endpoint as the treated biofilm [7, 9–11], assuming that the constitution (e.g. viable cell numbers, total biomass etc.) of the untreated reference growth control biofilm is stable from the time point of pre-antimicrobial exposure to the measured endpoint. Four theoretical scenarios showing the consequences of stable and unstable quantities of untreated reference biofilm viable cells (CFU/mL) over the course of the experiment for the interpretation of anti-biofilm effects are listed in Table 1. Provided the established biofilm had a starting quantity of 10^5^ CFU/mL before antimicrobial treatment (Table 1, scenario A), the quantification of 10^8^ CFU/mL of the untreated biofilm at the measured endpoint reveals that the biofilm without the addition of antibiotics increased by 3 log_10_ in CFU/mL. The treated biofilm with 10^5^ CFU/mL at the measured endpoint, however, implies no increase in the mean number of biofilm viable cells, making this scenario a classic example for the determination of inhibitory effects. The further growth of the biofilm was inhibited with addition of the antimicrobial substance by 3 log_10_ in CFU/mL in scenario A, but the biofilm was not reduced by 3 log_10_ in CFU/mL, which would be the conclusion if the untreated reference growth control biofilm is regarded only at the measured endpoint, but not before antimicrobial exposure. Scenario B illustrates an unstable quantity of the untreated reference biofilm over the course of the experiment as well, but in a smaller magnitude. If the untreated reference biofilm at the time point of pre-antimicrobial exposure and at the measured endpoint is composed of 10^7^ CFU/mL and 10^8^ CFU/mL, respectively, the untreated biofilm increased by 1 log_10_ CFU/mL in this time span. With the same readout of the treated biofilm at the measured endpoint of 10^5^ CFU/mL, this scenario indicates that the biofilm was reduced by 2 log_10_ in CFU/mL (from 10^7^ to 10^5^ CFU/mL) with the addition of the antimicrobial substance. Notably, only the starting quantity of the established, mature biofilm changed, but not the final results at the measured endpoint of this theoretical anti-biofilm assay. Only if the untreated reference biofilm is stable in CFU/mL numbers in the time span of pre-antimicrobial exposure and assay readout (scenario C), the interpretation of the anti-biofilm effect size (3 log_10_ reduction with a decrease of 10^8^ to 10^5^ CFU/mL) is the same when evaluating the effect based on the reference biofilm at the measured endpoint or the time point before antimicrobial exposure. If the untreated reference biofilm shows a higher viable cell quantity before antimicrobial exposure than at the time point of readout (scenario D), the decrease of viable cell numbers independent on antimicrobial treatment needs to be considered for the interpretation of the anti-biofilm effect size. In scenario D, this means the biofilm quantity decreased treatment-independent from 10^9^ CFU/mL to 10^8^ CFU/mL, leading to a 3 log_10_ reduction from 10^8^ to 10^5^ CFU/mL due to antimicrobial treatment. Above scenarios illustrate that only *after* the anti-biofilm experiment has been performed and, essentially, had included an assessment of the established biofilm before antimicrobial exposure, one can clearly say i) whether a biofilm reducing or inhibitory effect has taken place, ii) how high the magnitude of the analysed effect is. Researchers should therefore match the according biofilm susceptibility parameter to the observed effect based on the quantification of the reference growth control biofilm before and after treatment.

**Table 1:** Interpretation of the anti-biofilm effect size based on different scenarios of starting viable cell numbers before treatment

	At measured endpoint	Scenario A	Scenario B	Scenario C	Scenario D
		Pre-antimicrobial exposure	Pre- antimicrobial exposure	Pre- antimicrobial exposure	Pre- antimicrobial exposure
Untreated	10^8^ CFU/mL	10^5^ CFU/mL	10^7^ CFU/mL	10^8^ CFU/mL	10^9^ CFU/mL
Treated	10^5^ CFU/mL	10^5^ CFU/mL	10^7^ CFU/mL	10^8^ CFU/mL	10^9^ CFU/mL
Interpretation	3 log_10_ biofilm reduction	3 log_10_ inhibition of biofilm growth (MBIC)	2 log_10_ biofilm reduction	3 log_10_ biofilm reduction (MBEC or BBC)	3 log_10_ biofilm reduction (MBEC or BBC)

*MBIC* Minimal biofilm inhibitory concentration, *MBEC* Minimal biofilm eradication concentration, *BBC* Biofilm bactericidal concentration

**Fig. 2:**
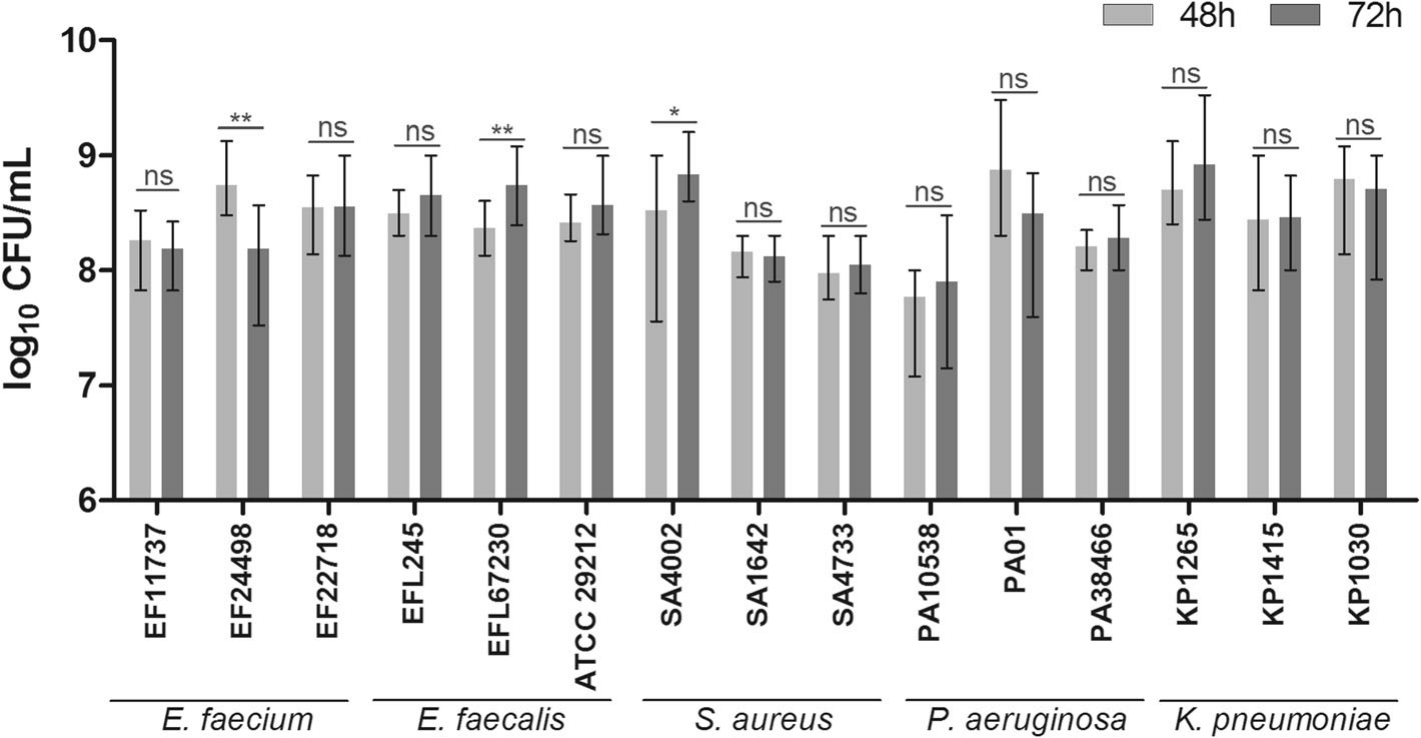
Biofilm viable cell numbers (CFU/mL) after 48 h and 72 h of growth. Shown are the mean values with ranges of triplicates. An unpaired t-test was performed to analyse significant differences (*P*-value < 0.05) between 48 h and 72 h of growth. ns = no significance

To assess how the starting number of biofilm viable cells may influence the interpretation of the anti-biofilm effects measured in our biofilm model, we determined the CFU/mL of untreated reference biofilms of five different bacterial species before and after potential antimicrobial exposure (Fig. 2). In our model, biofilms are grown for 48 h followed by incubation of antimicrobial substance for 24 h, resulting in 72 h of growth of untreated reference biofilms at the measured endpoint. Bacterial suspensions (0.5 McFarland) of three clinical isolates of each *Enterococcus faecium, Enterococcus faecalis, Staphylococcus aureus, Pseudomonas aeruginosa* and *Klebsiella pneumoniae* prepared in Müller Hinton broth or Todd Hewitt broth (both Karl Roth, Karlsruhe, Germany) for enterococci, respectively, were inoculated in triplicates in plastic microtiter plates (Greiner Bio-one, Frickenhausen, Germany). Biofilms were grown at 37 °C, 5% CO_2_ without shaking for 48 h and 72 h with change of medium after 48 h to mock antimicrobial treatment. After 48 h and 72 h, respectively, biofilms were washed, resuspended and selected 10-fold dilutions were plated for determination of CFU/mL. 80% of the tested isolates showed no significant increase in CFU between both time points (Fig. 2). For two strains (EFL67230 and SA4002), CFU_72h_ was significantly increased compared to CFU_48h_, however below 1 log_10_ and therefore not influencing the interpretation of the results in terms of reducing or inhibiting effects. One *E. faecium* isolate (EF24498) showed a significant decrease in CFU/mL, but again below 1 log_10_. The constancy of biofilm cell numbers from the time point of pre-antimicrobial exposure to the measured endpoint implies the determination of biofilm reducing instead of inhibiting effects in our model (Table 1, scenario C). A decrease in viable cell numbers of the treated biofilm compared to the untreated reference biofilm at the measured endpoint (which has the equal quantity as the untreated reference biofilm pre-antimicrobial exposure) can clearly be related to a reduction of the biofilm due to antimicrobial treatment. If viable biofilm cell numbers were increasing between 48 h and 72 h, either the effect magnitude of the reducing effect would change (Table 1, scenario B) or inhibiting instead of reducing effects would be analysed (Table 1, scenario A), making it necessary to determine the anti-biofilm effect size based on the quantification of the established, mature biofilm before antimicrobial treatment. Importantly, the constancy of the reference biofilm may not be the case for other methods of biofilm growth, e.g. dynamic biofilm reactors where biofilms are grown under constant nutrient flow [3], highlighting the importance of individual quantification of mature, pre-treatment biofilms for each biofilm model.

## Conclusions

Above scenarios elucidate another point of many current difficulties in biofilm methodology. Presently, biofilm susceptibility endpoint parameters are inconsistently perceived, used and interpreted among biofilm researchers. For example, Sandoe et al. quantified their peg biofilms before and after exposure to ampicillin, showing a significant reduction in CFU/mL numbers, yet using the MBIC as biofilm susceptibility endpoint parameter to describe their results [11]. To overcome this lack of consistency, standardized methods with accurate and precise definitions of biofilm susceptibility endpoint parameters are urgently needed, reducing confusion and contradictions among different anti-biofilm component studies. For the clinical evaluation of anti-biofilm compounds intended for therapy of BAI, it is crucial to determine whether a drug is able to penetrate and eradicate, in part or completely, the biofilm structure or is only able to inhibit its further growth.

The current insufficient evidence to recommend antibiotics on the basis of biofilm susceptibility testing is mainly attributed to the deficit of proper methodology representing in vivo biofilms [5]. The fact that the very few clinical trials addressing BAI have not measured biofilm eradicative but inhibitory effects might contribute to the poor observed correlation between biofilm susceptibility testing and clinical outcome [10–12]. Commercially available anti-biofilm test kits like the MBEC Assay®, formerly the Calgary Biofilm Device (Innovotech, Edmonton, Canada), show increasing rates of use in biofilm research [6], however neglect the potential problem of not measuring reducing, but inhibitory effects. Although the datasheet of the MBEC Assay® recommends a biofilm growth check before antimicrobial treatment [13], most publications do not take those values into account for the interpretation of anti-biofilm effects [10–12, 14]. We therefore highly encourage biofilm researchers to assess established biofilms before antimicrobial exposure, independent on the method of choice for biofilm growth and assay readout, to bring more clarity to their measured biofilm effect size and biofilm susceptibility parameters. The assessment of pre-treatment biofilms will contribute to a standardized use of biofilm susceptibility endpoint parameters, which is urgently needed to improve the comparability of anti-biofilm studies and to make progress in the development of clinically meaningful anti-biofilm assays.

## Data Availability

All data generated or analysed during this study are included in this published article.

## Abbreviations

BAI: Biofilm-associated infection
BBC: Biofilm bactericidal concentration
BPC: Biofilm prevention concentration
CFU: Colony forming unit
CLSI: Clinical & Laboratory Standards Institute
EUCAST: European Committee on Antimicrobial Susceptibility Testing
MBEC: Minimal biofilm eradication concentration
MBIC: Minimal biofilm inhibitory concentration
MIC: Minimal inhibitory concentration
